# Trajectories and strategies in implementing screening, brief intervention, and referral to treatment for substance use in primary care within public hospitals: a longitudinal qualitative study

**DOI:** 10.1186/s43058-025-00796-9

**Published:** 2025-11-12

**Authors:** Lina Tieu, Elizabeth Bromley, Rajat Simhan, Roshan Bastani, Beth A. Glenn, Nadereh Pourat

**Affiliations:** 1 Department of Health Policy and Management, UCLA Fielding School of Public Health, Los Angeles, CA USA; 2 Center for Healthcare Policy and Research, University of California, Davis, 4900 Broadway, Suite 1430, Sacramento, CA 95817 USA; 3 Department of Psychiatry and Biobehavioral Sciences, University of California, Los Angeles, CA USA; 4 San Joaquin Health Centers, French Camp, California, USA; 5 Center for Cancer Prevention and Control Research and UCLA-Kaiser Permanente Center for Health Equity, Fielding School of Public Health, Jonsson Comprehensive Cancer Center, UCLA, Los Angeles, CA USA; 6 UCLA Center for Health Policy Research, Los Angeles, CA USA

**Keywords:** Alcohol, Substance use, Implementation, Screening, brief intervention, and referral to treatment (SBIRT), Public hospitals, Primary care

## Abstract

**Background:**

Screening, brief intervention, and referral to treatment (SBIRT) is an evidence-based approach to identify and initiate treatment for alcohol and substance use in primary care settings. Among 22 public hospitals incentivized to implement SBIRT as part of a value-based Medicaid waiver program over five years, this study examined trajectories, strategies, and challenges in standardizing SBIRT within primary care.

**Methods:**

This study utilized data from narrative reports completed by hospital leadership, obtained from the evaluation of the Public Hospital Redesign and Incentives in Medi-Cal (PRIME) program in California. Following the Multi-Level Health Outcomes Framework, template analysis was used to characterize SBIRT implementation. Content analysis was used to catalogue implementation strategies using the Expert Recommendations for Implementing Change Framework. To assess trajectories (i.e., longitudinal implementation outcomes) of SBIRT implementation, we categorized standardized adoption of sequential SBIRT processes (screening only; screening and brief intervention; screening, brief intervention, and referral to treatment) and reach (limited vs. full primary care population).

**Results:**

Hospitals used a wide variety of measures, personnel, platforms, and workflows in screening for substance use within primary care settings. Brief intervention was conducted by primary care or behavioral health care team members who had received targeted training. Hospitals implemented a wide range of treatment options to address substance use, including referral to co-located or contracted/partnered behavioral health providers. By the end of the first implementation year, only one hospital had standardized screening processes, and none had standardized brief intervention or referral. At the end of the fifth year, 20 of 22 hospitals had standardized screening, 15 had standardized brief intervention, and 12 had standardized referral among their full primary care populations. Strategies and challenges in planning, education, and restructuring processes (e.g., integration of screening processes within electronic health records and clinical workflows) were particularly influential in facilitating implementation.

**Conclusions:**

This study highlighted significant progress made by public hospitals in implementing standardized SBIRT processes among their primary care populations within a value-based program. However, hospitals experienced delays and challenges, highlighting key areas in which additional support or investment may be needed to sustain and promote long-term progress in SBIRT implementation.

Contributions to the literature

- Screening, brief intervention, and referral to treatment (SBIRT) is recommended for addressing alcohol and substance use in healthcare settings. Knowledge about influences, strategies, and trajectories for implementing SBIRT can promote more comprehensive and timely implementation.
- This study is innovative in examining the longitudinal trajectories (i.e., implementation outcomes over time) of SBIRT implementation in primary care settings over a five-year period and within a comprehensive value-based program focused on improving ambulatory care.
- Influences and strategies for SBIRT implementation are highlighted among a varied sample of public hospitals that serve as an important source of care for individuals among the most vulnerable to social disadvantages and chronic and acute health conditions.

## Background

Substance and alcohol use are leading preventable causes of morbidity and mortality [1–4]. Increasingly, there are efforts to promote earlier identification and treatment of unhealthy alcohol and substance use within the general population through the implementation of evidence-based strategies within primary care settings [5, 6]. Screening, brief intervention, and referral to treatment (SBIRT) has been shown to be an effective tool to identify and initiate treatment for unhealthy alcohol use in clinical and primary care settings [5–14]. SBIRT is a comprehensive approach consisting of three steps: (1) use of a standardized screening measure to determine the severity of unhealthy substance use; (2) among patients screening positive for unhealthy substance use, brief intervention such as motivational interviewing or counseling to promote awareness and motivation for behavioral change; and (3) among patients with higher severity of unhealthy substance use and related consequences, referral to specialty care treatment [15, 16]. The United States Preventive Services Task Force (USPSTF) has recommended the use of screening and counseling for unhealthy alcohol use within primary care settings since 1996 and for drug use since 2020 [17, 18].

Strategies for implementing SBIRT have been examined within a variety of health settings, including community health centers; integrated health care systems; school-based health centers; and specialty, acute care, and emergency departments [19–23]. Prior research has examined the implementation of SBIRT within systems participating in dedicated initiatives, including programs supported by the Substance Abuse and Mental Health Services Administration and Agency for Healthcare Research and Quality, as well as state-run programs to promote SBIRT implementation [7, 24–28]. Such studies have identified considerable variability in best practices and strategies commonly employed in the implementation of SBIRT, including supporting multidisciplinary care team members in carrying out the steps of SBIRT, establishing leadership support, establishing referral networks, and electronic health record (EHR) integration [19, 23, 24, 29]. However, there is a dearth of literature that examines strategies for SBIRT among public hospital systems that serve as an important source of care for patient populations that often face a high burden of structural disadvantage and health needs, and studies that examine the pace of implementation of SBIRT within primary care settings [30].

From 2015–2020, California’s public hospitals participated in the Public Hospital Redesign & Incentives in Medi-Cal (PRIME) Program, a Medicaid Sect. 1115 waiver program (which confers flexibility to states in implementing novel approaches within their Medicaid programs) that provided up to $7.5 billion in federal and state funding to transform and improve access to ambulatory care [31, 32]. In addition to providing acute care, public hospitals are safety net providers with dedicated clinics providing comprehensive primary care services [33]. Under PRIME, hospitals participated in up to 18 required and elective projects focused on improving delivery of outpatient care (e.g., primary care redesign) and care for targeted populations (e.g., chronic pain management), with pay-for-reporting and pay-for-performance incentives tied to bi-annual performance on specific metrics within each project over a five-year period [31, 34]. Among these initiatives, 22 public hospitals participated in a project to improve the integration of behavioral health care within their primary care settings, which included an SBIRT-specific metric aimed at increasing SBIRT rates among primary care patients aged 12 and older [34–36]. Activities to support hospitals in implementing PRIME projects included guidance on recommended core components of implementation, participation in learning collaboratives (including those specific to mental health, health disparities, and substance use disorders), guidance from metric stewards, and annual PRIME conferences [34]. Such activities were designed to promote dissemination of recommended practices while providing substantial flexibility to hospitals in identifying and implementing specific strategies and processes for SBIRT.

This study examined data from hospitals participating in the PRIME program to (1) describe trajectories (i.e., longitudinal implementation outcomes) of SBIRT implementation in primary care settings over a five-year period, and (2) describe influences, strategies, and challenges to systematically implementing SBIRT in primary care settings.

## Methods

### Sample and study setting

SBIRT implementation was examined within 22 public hospitals in California: (1) twelve County-operated designated public hospital (DPH) systems that are concentrated in densely populated, urban regions and predominantly serve patients who are Medicaid beneficiaries or are uninsured; (2) five University of California (UC) designated public hospital systems that predominantly serve individuals with higher complexity; and (3) five district and municipal public hospital systems (DMPH) predominantly set in less densely populated or rural settings, with varying payer and case mix. Characteristics of the sample are presented in Table 1.

**Table 1:** Hospital characteristics

	**County-operated DPH** **N = 12**	**University of California DPH** **N = 5**	**DMPH** **N = 5**
**Characteristic, mean (range)**			
Primary Care Facilities	11.5 (1–32)	20.8 (8–60)	2.2 (1–4)
Hospitals	1.8 (1–5)	1.8 (1–3)	1 (1–1)
Annual Visits	475,567 (132,000–1,180,600)	825,720 (504,300–1,048,300)	63,700 (19,500–185,000)
Proportion of Primary Care Population with Medicaid Managed Care	62% (39–87%)	10% (0.6–24%)	n/a
Case Mix	1.2 (1.0–1.3)	1.8 (1.7–2.0)	1.1 (1.0–1.3)

*DPH *designated public hospital, *DMPH *district and municipal public hospital

### Conceptual frameworks

#### Multi-level health outcomes framework

**Fig. 1:**
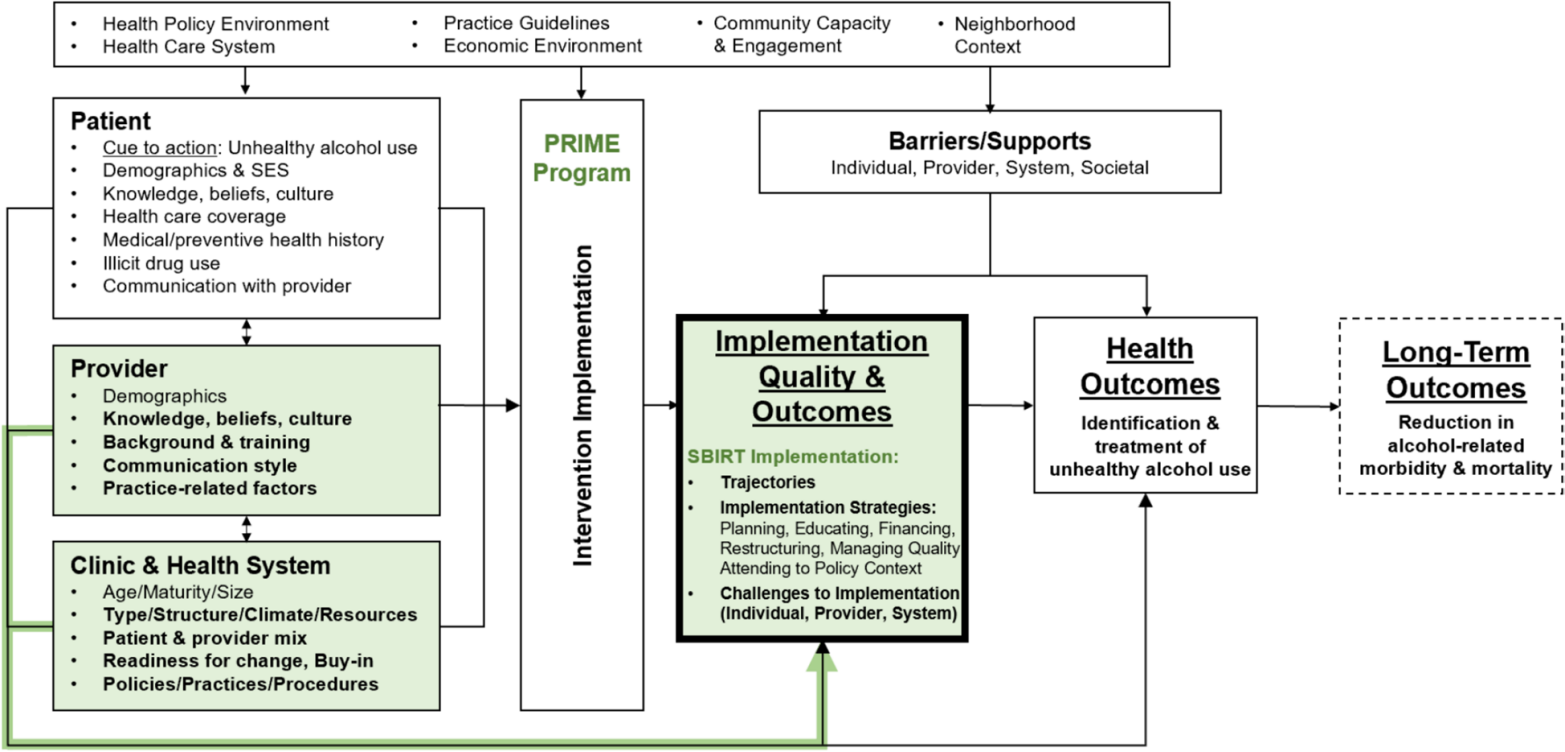
Multi-Level Health Outcomes Framework. PRIME: Public Hospital Redesign and Incentives in Medi-Cal; SBIRT: screening, brief intervention, and referral to treatment. Adapted from Bastani, et al., UCLA, 1990–2022 [37–43]

This study utilized the Multi-Level Health Outcomes Framework (MHOF), a multi-level, socioecological model that combines elements of conceptual formulations related to the implementation of interventions to improve health behaviors and outcomes to guide thematic analysis (Fig. 1) [44–51]. Given our interest in assessing multi-level factors that may influence SBIRT implementation, the MHOF provided an initial framework for identifying a priori themes to describe and assess the strategies utilized by public hospitals in implementing SBIRT, with a special focus on factors at the provider/staff and clinic/health system levels [37]. For example, provider-level factors influencing SBIRT implementation included training of providers to carry out SBIRT processes, and clinic/health system factors included tools and measures used to screen patients for substance use. In particular, we used the MHOF to theorize both immutable (e.g., patient characteristics) and mutable (e.g., provider knowledge of SBIRT best practices) factors likely to influence utilized strategies and quality of SBIRT implementation within public hospital settings.

#### Expert recommendations for implementing change framework

Following thematic analysis guided by the MHOF, the Expert Recommendations for Implementing Change (ERIC) framework was referenced to organize the strategies hospitals used to implement SBIRT into 6 categories: (1) planning, (2) educating, (3) financing, (4) restructuring, (5) managing quality, and (6) attending to policy context [52, 53]. The ERIC framework has been applied to prior research describing the implementation of behavioral health interventions, including SBIRT within acute care settings [20].

### Data sources and measures

This study leveraged data obtained from a statewide evaluation of the PRIME program conducted by Pourat, et al. in 2015–2022 [34–36]. As part of requirements for PRIME monitoring and reporting to the CA Department of Health Care Services, leaders at each hospital, specifically those involved in overseeing and carrying out the implementation of PRIME activities at each timepoint, were required to complete narrative progress reports to characterize their PRIME project activities from July 2015 to June 2020. Narrative reports were completed bi-annually, the first of which was completed at the end of the first year, and subsequent reports occurring every 6 months (at the mid-year and year-end timepoints) in Years 2–5. Thus, the data used in this study included 9 narrative reports per hospital (198 in total). Narrative reports followed a structured template covering all PRIME activities implemented by the hospitals. Specific to this study, the narrative reports contained free response fields related to the following: definition and methodology for determining target population for PRIME metrics; participation in grant-funded initiatives or learning collaboratives; plans to sustain and build on progress in implementing behavioral health integration for the remainder of the program; SBIRT data capture methods, challenges, and resolutions; and SBIRT quality improvement efforts, challenges, and resolutions. Reports were approximately 100 pages each and were reviewed by the CA Department of Health Care Services for completeness at each interval.

### Analysis

The analyses in this study focused on uncovering commonalities and variation in influences, strategies, and trajectories of public hospitals in implementing SBIRT [54].

#### SBIRT trajectories and implementation outcomes

To examine trajectories (i.e., longitudinal implementation outcomes) of SBIRT implementation within each hospital’s primary care population over a five-year period, one member of the research team (LT) examined all project narratives and drafted summaries of and pulled passages relating to all activities involving SBIRT implementation reported by each hospital at each timepoint. Two coders (LT, EB) with graduate-level training in qualitative analysis and prior experience in behavioral health research independently reviewed the narrative data at each timepoint and assigned a status of standardized (vs. ad hoc) SBIRT adoption that had been reached at the end of each year for each hospital among their primary care population (Fig. 1). SBIRT status differentiated between adoption of standardized processes (i.e., indicating intentional implementation of standard SBIRT processes) for each sequential step of SBIRT (i.e., screening only; screening and brief intervention; screening, brief intervention, and referral to treatment) and reach (i.e., narrative indicating implementation within a limited vs. full primary care population). Inter-rater reliability was examined for ratings of SBIRT adoption and reach across all timepoints by the two independent coders. Discrepancies were discussed and reconciled to create the final specification of implementation outcomes. Outcomes were examined separately for County-operated DPHs (*n* = 12), University of California DPHs (*n* = 5), and DMPHs (*n* = 5) to identify potential differences in implementation timelines by hospital type.

#### SBIRT strategies

As a first step to assessing thematic findings related to SBIRT implementation, template analysis methodology was used to identify hospital implementation processes. Template analysis allowed for the use of a priori themes based on factors identified within the MHOF model and prior literature [19, 23, 24, 29], with flexibility to adapt the analysis framework to include emerging themes [55]. The initial analysis framework included a priori themes within specific categories of the MHOF (Provider, Clinic & Health System, Implementation Quality & Outcomes) anticipated to play a salient role in describing or influencing SBIRT implementation. For example, a priori themes included communication between care team members (Provider), staffing models (Clinic & Health System), and infrastructure created to support SBIRT (Implementation Quality & Outcomes) [37].

Analysis of the narrative reports was used to describe phases of implementation progress and to identify markers of success within each phase. One coder (LT) analyzed narrative reports from the initial two time periods (conducted at the end of the first year and midpoint of second year of implementation) and established an initial codebook. Following discussion of emerging themes, the codebook was reviewed and refined by three additional team members (EB, BG, NP) before being applied to the remainder of the narrative reports by one coder (LT).

Subsequently, content analysis was applied to catalogue the SBIRT strategies used by hospitals according to the six categories within the ERIC framework [52, 53]. This step of the analysis focused on describing the key activities used, and barriers and facilitators encountered by hospitals in implementing SBIRT and assessing the intensity of efforts within different categories of implementation. Qualitative analysis of narratives was conducted using Atlas.ti (version 22.1.5.0).

## Results

### Trajectories of SBIRT Implementation

The inter-rater reliability of assessment of SBIRT status (standardized screening; standardized screening and brief intervention; standardized screening, brief intervention, and referral) and reach (standardized among limited population vs. full primary care population) indicated an average joint probability of agreement of 85%.

By the end of the program (i.e., end of fifth year of implementation), 7 of 12 County-operated DPHs, one of five UC DPHs, and four of five DMPHs noted that they had implemented standardized processes for screening, brief intervention, and referral within their full primary care populations. Three County-operated DPHs, two UC DPHs, and one DMPH (27% of all hospitals) had not yet moved beyond the process of implementing standardized screening processes by the end of the fifth year.

**Fig. 2:**
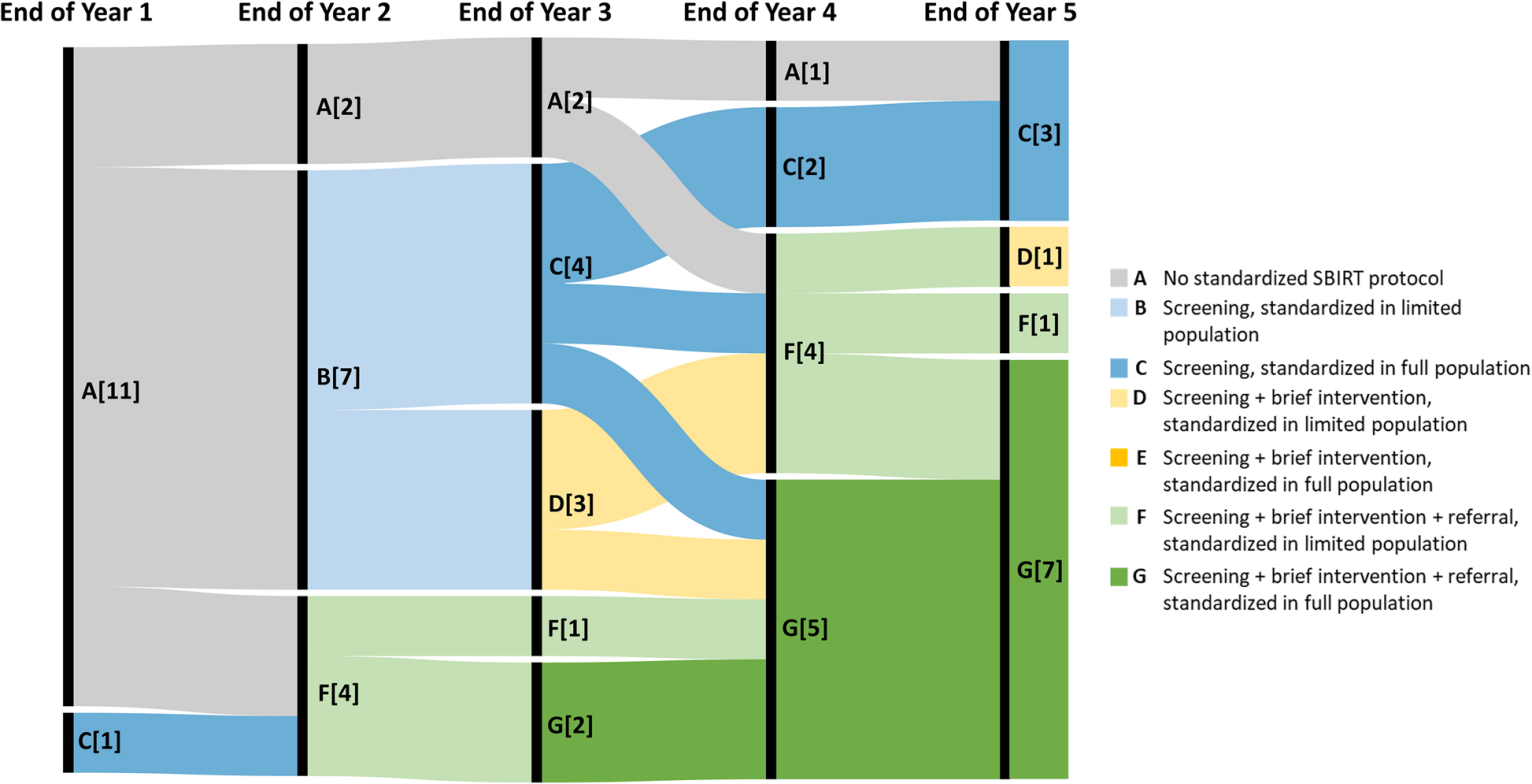
Trajectories of SBIRT Implementation among County Designated Public Hospitals, *n* = 12. Note: Numbers represent the number of hospitals at each SBIRT status at the end of the implementation year

Among County-operated DPHs, trajectories revealed gradual progress in implementing the sequential steps of SBIRT in each year (Fig. 2). Specifically, one County-operated DPH had implemented standardized screening, while all others described having no standardized SBIRT protocol at the end of the first year. At the end of the second year, the majority of County-operated DPHs described having implemented standardized processes for screening (*n* = 7) or referral (*n* = 4) within a subset of their primary care populations. The end of Year 3 saw the first County-operated DPHs (*n* = 2) implement standardized processes for all SBIRT steps among their full primary care population, with a greater proportion having implemented standardized SBIRT within a limited (*n* = 4) or full (*n* = 5) population by the end of the fourth year.

**Fig. 3:**
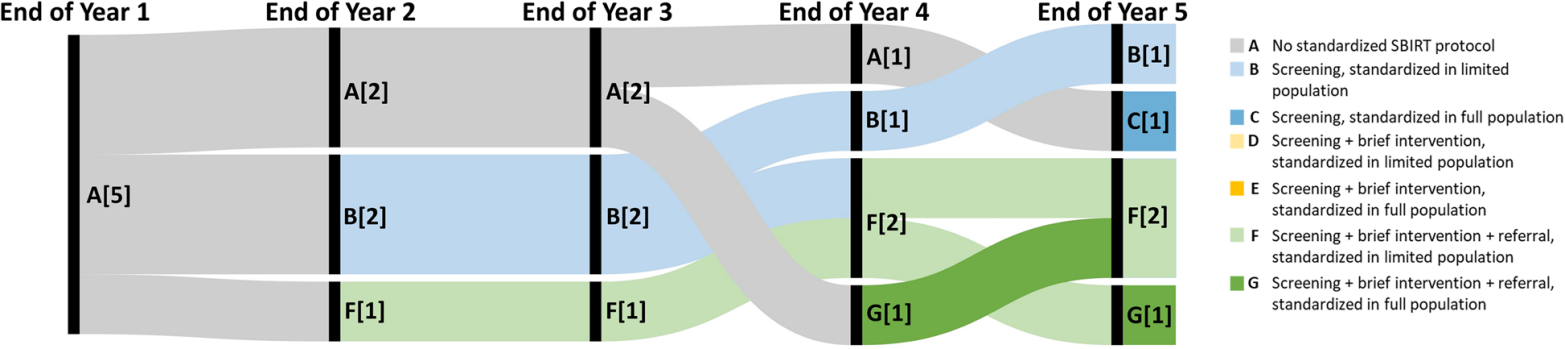
Trajectories of SBIRT Implementation among University of California Designated Public Hospitals, *n* = 5. Note. Numbers in brackets represent the number of hospitals at each SBIRT status at the end of the implementation year

**Fig. 4:**
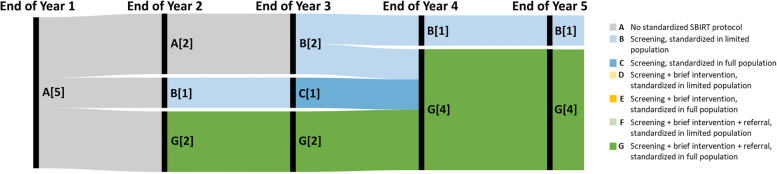
Trajectories of SBIRT Implementation among District and Municipal Public Hospitals, *n* = 5. Note. Numbers represent the number of hospitals at each SBIRT status at the end of the implementation year

Among UC DPHs, no hospitals had implemented any standardized SBIRT processes until the end of the second year (Fig. 3). Trajectories revealed that rollout of SBIRT processes often occurred among limited populations. DMPHs noted more rapid standardization of SBIRT (Fig. 4). Among the five DMPHs, two had standardized each SBIRT process among their full primary care population by the end of the second year of implementation. By the end of the fourth implementation year, 4 noted having implemented standardized processes for all SBIRT steps among their full populations, while one hospital noted being unable to move beyond standardized screening among a limited population.

Across hospitals, the most progress was achieved in the third and fourth years of PRIME implementation, with many hospitals moving beyond screening to deliver brief interventions. Notable trajectories included one UC DPH that progressed from having no standardized SBIRT protocols in the third year to having standardized each SBIRT step by fourth year of implementation. This hospital noted that templates for documenting substance use via brief (e.g., one-item) and full screening tools as well as referral/treatment options were integrated within the EHR in the fourth year of implementation, which allowed them to transition from ad hoc to standardized SBIRT implementation. In the following year, this hospital implemented brief screening via online questionnaires, with full screens triggered on an as-needed basis to determine the need for intervention. For the four DPHs who did not progress beyond standardized screening by the end of the program, challenges to SBIRT implementation included major delays in integrating substance use screening into the EHR, large geographic spread of primary care clinics, and limited availability of substance use treatment. Among DMPHs, one hospital that was not able to move beyond the standardized screening process noted provider and staff attrition and difficulty linking patients to care at external facilities.

### SBIRT components implemented by hospitals

Specific SBIRT components implemented by hospitals and illustrative quotes are outlined in Table 2 and discussed below.

**Table 2:** Processes Used for Screening, Brief Intervention, and Referral to Treatment

SBIRT Process	Implementation Examples	Illustrative Quote(s)
**Screening**		
Screening measures used to assess substance use	• One-item substance use screen • Alcohol Use Disorders Identification Test (AUDIT)/AUDIT-Concise (AUDIT-C) • CAGE (Cut down, Annoyed, Guilty, Eye-opener) Questionnaire/CAGE-AID (CAGE Questions Adapted to Include Drugs) • Drug Abuse Screening Test (DAST)/DAST-10 • Staying Healthy Assessment (for adolescents) • CRAFFT (Car, Relax, Alone, Forget, Friends, Trouble; for adolescents, young adults) • TeenScreen (for adolescents) • Screening to Brief Intervention (S2BI; for adolescents) • 4Ps Plus, 5Ps (for pregnant individuals)	Given the complexity of creating standard work across [multiple] clinics in the context of many other projects and demands, we are working toward developing an integrated screening tool … As conceptualized, this tool will incorporate screening for depression, smoking, substance use, and intimate partner violence (IPV). (Hospital 8, Year 2 Midyear) If a patient scores positive to the initial screen (brief screen) about alcohol and drug use, they are then assessed for alcohol and/or drug use severity using the CAGE questionnaire. (Hospital 9, Year 4 Midyear)
Platforms piloted or implemented for administration of substance use screening	• EHR-integrated templates, modules, or reports • Paper surveys • Patient portal survey • Electronic survey tools used at point of care (e.g., tablet-based)	Tablets purchased to create ease for the patient do not work. Due to several challenges, it is unlikely that we will be able to utilize an electronic screening tool that populates the patient’s chart … This is due to a host of technological issues with no resolution in sight. (Hospital 10, Year 2 Year-end) [Hospital] has implemented electronic survey administration software which allows survey results to be captured and recorded immediately … this software contains the ability to track frequency of surveys administered, and after administration, instantly notify the care team of any flags identified. A challenge for this metric is that we are still in the process of rolling out our survey administration platform in all clinics, and some of our clinics are still utilizing paper surveys. (Hospital 4, Year 3 Year-end) The SBIRT questionnaire is now a part of patient intake workflow and results are being recorded electronically within the [Vendor] EHR system. (Hospital 9, Year 3 Year-end) After much effort, [EHR Vendor] Portal teams have determined functional configurations to implement preencounter SBIRT screening of patients using [pre-visit] questionnaires and are working to implement in clinical practice. (Hospital 14, Year 5 Year-end)
Staff responsible for screening	• Medical Assistant • Nurse • Primary care provider • Registration/front desk staff	PHQ-9, SBIRT and CRAFFT screening tools have been developed as assessments in the EMR and are currently being utilized by all clinic nursing staff. Staff was properly trained on the data entry, as well as providers on referencing the results of this screening and initiating referrals based upon scores as indicated. (Hospital 18, Year 2 Year-end) All primary care clinics have implemented behavioral health screening for literate adult English and Spanish speaking patients. Pre-screening & follow-up screening are performed at registration, data is entered in the EHR by the MA at intake, and results present in provider workflows for their action. (Hospital 1, Year 3 Year-end) Work flows have been established in our primary care clinics for back office staff to do a brief annual questionnaire. If the results are positive, providers are alerted to perform the full screening and take action if necessary. (Hospital 13, Year 4 Year-end)
Screening workflows	• Use of reports to identify patients in need of screening • Staff outreach to patients due for screening • Integration into patient intake/rooming workflows • Administration of screening during medical visit • Documenting screening with EHR templates • Use of procedure codes to bill for screening • Electronic scoring of screens and clinical decision support	[The hospital] has acquired electronic survey administration software which allows screenings to be administered in a non-intrusive manner, and also allows for subsequent questions and screenings to be tailored to responses from the individual. This allows for the capability of properly scoring SBIRT surveys … which in turn provides for enhanced, personalized care. (Hospital 4, Year 2 Midyear) There are features within [population health platform], such as the morning huddle report, which show when a patient is due for screenings (i.e., PHQ-9 and SBIRT). We are currently using this mechanism on a daily basis and fine-tuning and verifying that the information flowing to this report is up-to-date and accurately used. (Hospital 18, Year 5 Year-end)
**Brief Intervention**		
Staff responsible for brief intervention	• Primary care provider • Registered nurse • Family medicine nurse • Medical assistant • Substance use counselor • Licensed clinical social worker (LCSW)	After implementing systematic alcohol and drug screening, our biggest challenge is developing a program to train staff to provide appropriate brief intervention for patients who have a positive screen. To minimize the burden to providers, we have identified bilingual clinic staff who can perform the brief intervention under the supervision of the provider. (Hospital 12 County, Year 2 Mid-year) … primary care teams now are able to provide a warm handoff to the embedded LCSW who provides brief intervention for patients with mild to moderate issues and refers for further treatment as needed. (Hospital 9, Year 2 Year-end) The [Clinic] care team is working together to standardize the screening, brief intervention, and referral to treatment for patients identified with a substance about issue. Medical Assistant[s] who are trained as Health Coaches are being utilized to conduct the brief intervention when necessary. (Hospital 11, Year 4 Midyear)
Processes for brief intervention	• Provider conversation or advice • Motivational interviewing, counseling, or coaching • Provide literature or educational materials	Through a coordinated care, team approach, the [targeted behavioral health program] team provides outreach and engagement to the identified patients, performs screening and follow up/counseling, supports self-management skills, facilitates timely and effective communication between the patients care providers all in an effort to manage not only the individuals behavioral conditions, but to make improvements with their medical conditions. Because of their expertise, and motivational interviewing skills, we decided it best to begin the SBIRT, alcohol and drug misuse screening and necessary follow up with these embedded teams. (Hospital 2, Year 4 Midyear) [The] Substance Abuse Counselor offers to meet with patients, [and] offers intervention using motivational interviewing with the goal of patient being open to services at [a] local substance abuse treatment agency. (Hospital 20, Year 4 Year-end)
**Referral to Treatment**		
Referral processes from primary care to provider of substance use treatment	• Warm handoffs to co-located behavioral health providers • Electronic referral system • Clinical decision support tool • Care coordination to connect patients to community sources of treatment	Facing challenges with availability of behavioral health services and successful referrals, the [County Health Agency] has worked to protocolize the referral process through clinical decision tools attached to the screenings and to automatically generate tasks and referrals where possible. (Hospital 12, Year 2 Year-end) In Jan. 2019, we launched a universal behavioral health referral process that streamlined six referral processes into one. The new process triages referrals to internal and external providers for faster response. (Hospital 3, Year 4 Year-end) Although still limited by few local resources for this purpose, we continue to develop relationships with county and local private entities to be able to refer patients for the help that they need in this arena. (Hospital 18, Year 5 Midyear)
Provider, staff, or site available for referral or treatment	• Substance use hotline • Behavioral health providers within the hospital (e.g., substance use counselor, licensed clinical social worker, psychiatrist) • Inpatient care • External behavioral health providers (e.g., contracted health plan, County or local behavioral health program, community health center)	Our Behavioral Health Program Manager has identified County Mental Health staff and is currently working on collaboration regarding standardized care and treatment plans. (Hospital 18, Year 2 Midyear) The [Hospital] Substance Use Disorder Treatment Clinic is part of the [Hospital] Behavioral Health Clinic. Staffed by an addiction psychiatrist and an LVN [licensed vocational nurse] with part-time support from social work and pharmacy, it started operations in Fall 2017 and will become fully operational in March 2018. It provides outpatient services … for adults, on a referral or facilitated referral basis. (Hospital 13, Year 3 Midyear)
Substance use care coordination processes	• Use of registries to manage care for patients with behavioral health and substance use issues • Medication management • Collaboration with county health and mental health divisions on treatment plans	… the PRIME Care Coordinator … [is a] member of a collaborative care team that includes primary care provider, psychiatrist, behavioral health team members, utilization review, clinical staff, and PRIME Project Manager. The Care Coordinator will develop treatment plans, facilitate coordination of treatment, provide necessary follow-up, education, and referrals in order to complement care. (Hospital 20, Year 2 Year-end) With the addition of community services assistants, [Hospital] has now expanded to quad teams [known] as Health Home Complex Care Teams. These new team members, similar to promotoras, provide outreach to patients in need of supportive assistance in connecting to behavioral health, substance abuse or primary care services in the community. They are already proving helpful in managing and improving their patients mental, physical, and social well-being. (Hospital 7, Year 4 Midyear) We have made enhancements to our EMR [electronic medical record] to enable documentation of refusals for AUDIT/DAST/CRAFFT. We developed a registry to track positive screens and patient response to them. We are working to incorporate further enhancements on the registry into the EHR [electronic health record]. (Hospital 16, Year 4 Midyear)

#### Screening

Hospitals used a wide variety of measures to assess substance use in primary care during PRIME, most commonly the Alcohol Use Disorders Identification Test (AUDIT); Cutting Down, Annoyance by Criticism, Guilty Feeling, Eye-Openers (CAGE) Assessment; Drug Abuse Screening Test (DAST); and adaptations of these tools (e.g., AUDIT-Condensed [AUDIT-C]). While many hospitals noted the use of a brief (e.g., one-item) screen followed by a full screening in screen-positive patients, others opted to implement full screening as the first step. A few hospitals described coupling substance use screening measures with other assessments (e.g., social history, depression, interpersonal violence) to enhance efficiency. For the majority of hospitals, primary care providers or other medical staff (e.g., nurse, medical assistant) were responsible for administering brief and full alcohol and drug use screens during the medical visit or rooming process, with a minority noting that front desk staff members screened during registration. These processes required systematic documentation using EHR templates, replacing ad hoc processes of documentation (e.g., patient notes, social history). Within a few hospitals, EHR templates automatically scored screens and triggered clinical decision support to guide next steps*.* A few hospitals discussed piloting other strategies to assess substance use during PRIME, including patient-facing electronic survey tools administered at the point of care or via patient portal, but described experiencing challenges to routine adoption.

#### Brief intervention

Documentation of brief intervention processes was less comprehensive than that for screening processes. Most often in the narratives, hospitals stated that the role of conducting brief intervention fell on primary care team members (e.g., primary care provider, nurse, medical assistant) who had received specific training. In other hospitals, co-located behavioral health teams or providers (e.g., licensed clinical social workers) delivered brief intervention. One hospital described having a dedicated substance use counselor in this role. Brief intervention strategies included provider-prompted conversations; motivational interviewing, counseling, or coaching techniques; and providing educational materials about substance use harm.

#### Referral to treatment

For patients requiring further treatment approaches, referral included warm handoffs to co-located behavioral health providers, use of electronic referrals, referrals aided by clinical decision support systems, and care coordinators to connect patients to treatment services (e.g., substance use hotlines, inpatient treatment settings, community providers). In addition to supporting patients to initiate treatment, some hospitals used patient registries to audit and coordinate care for patients with documented substance use and collaborated with external providers to develop treatment plans. Substance use treatment partners included contracted providers (e.g., behavioral health plans), formal partnerships with County or local behavioral health programs, or informal relationships with other providers.

### Strategies and challenges in implementing SBIRT

Aligned with the major categories of the ERIC framework, prominent strategies (i.e., methods used to promote implementation) and challenges (i.e., factors hindering or delaying implementation) to implementing standardized SBIRT processes that emerged from hospital narratives are discussed below.

#### Planning

Many hospitals reported a previous focus on improving screening and treatment of depression, which was leveraged to implement SBIRT workflows. To select specific SBIRT strategies, some hospitals piloted strategies among a subset of patients that were easier to reach with existing resources (e.g., patients with English preferred language, patients of a small subset of clinics or providers) or for whom SBIRT implementation may have higher urgency (e.g., patients with multiple or severe medical and behavioral health conditions). Piloting workflows allowed hospitals to assess feasibility and incorporate feedback on SBIRT processes before spreading processes more widely:

*Thoughtful rollout of the [combined health and substance use] Screen required established improvement methodologies such as testing the tool with two providers; soliciting feedback; and incorporating changes prior to clinic-wide implementation. This strategy will ensure long-term acceptance and sustainability of the [screening tool] into workflows.* (Hospital 3, Year 2 Year-end)

The leadership that guided SBIRT implementation varied in focus (general behavioral health integration vs. SBIRT-specific) and staffing models (e.g., SBIRT manager vs. multidisciplinary team working to lead SBIRT implementation):

*A multi-disciplinary team (addictionologist, psychiatry, primary care, pain management) has been formed to discuss and choose a standardized evidence-based SBIRT screening tool.* (Hospital 13, Year 2 Midyear)

Strategies reported as being helpful for promoting buy-in for SBIRT implementation within systems included training to execute SBIRT workflows; explaining the importance of SBIRT within the wider context and goals of the PRIME program; and addressing the stigma of substance use (e.g., “reframing addiction as a chronic disease”; Hospital 10, Year 4 Midyear). Challenges to establishing buy-in among providers and staff included competing demands; discomfort among providers and staff with discussing alcohol and substance use with patients; and lack of treatment capacity for patients who screen positive. In particular, lack of treatment capacity was more prominently discussed among County-operated DPHs with large patient populations and in earlier years of implementation. To address this challenge, hospitals discussed the importance of developing relationships between primary care and specialty treatment providers for substance use through co-location; multidisciplinary care teams; and formal and informal partnerships with external specialists, clinics, and inpatient treatment programs.

#### Education

Intensive training efforts were required to prepare providers and staff to implement SBIRT workflows. The availability of existing resources enhanced perceived capacity for SBIRT training. Training materials were developed by hospitals or external consultants, and included protocols, best practice curricula, training videos, and print materials such as one-page handouts:

*We created quick reference sheets, which we call one-pagers to better assist staff and providers with understanding key areas of care, documentation, and coordination. These one-pagers serve as teaching guides for in-person trainings and discussions as well as clinic-level dissemination to direct care staff and providers.* (Hospital 12, Year 4 Midyear)

Training was disseminated via dedicated in-service trainings or presentations, regular provider/staff or quality improvement meetings, individual coaching, and train-the-trainer models. While hospitals did not describe conducting direct assessments of practice change following such trainings, several hospitals reported that tests of knowledge or competency were administered:

*The last training that was provided … on Motivational interviewing techniques. There was a 9% increase on the Pre/Post Test provided to staff.* (Hospital 20, Year 2 Year-end)

Some hospitals reported the acquisition of specific funding to support training efforts. In addition, partnerships with external organizations (e.g., academic medical centers, learning collaboratives) were leveraged to guide SBIRT training efforts.

#### Financing

PRIME tied incentive payments to the successful achievement of a designated SBIRT metric each year. In the fourth year of PRIME, the specifications for the SBIRT metric shifted from one combined metric measuring receipt of screening, brief intervention or referral to two separate metrics: (1) receipt of brief annual screen, and (2) receipt of full screen, brief intervention, and referral to treatment. This complicated implementation for hospitals who required changes to their documentation and reporting processes to meet evolving requirements for demonstrating achievement of the metric. Both DPHs and DMPHs described pursuing other funding to hire behavioral health staff or support SBIRT implementation:

*This grant will fund the addition of an Alcohol and Drug Treatment Specialist, 3 medication refrigerators for our clinics that will hold supplies of Narcan, Vivitrol, and Suboxone, and bus tickets for patients.* (Hospital 12, Year 4 Midyear)

A few hospitals discussed internal funding mechanisms (e.g., pay differentials or pay-for-performance) to incentivize providers and staff to complete SBIRT training or implement SBIRT strategies:

*Nurses can now earn a small pay differential for achieving SBIRT certification through the 4-hour course.* (Hospital 12, Year 3 Midyear)

*We incentivized clinics to complete [behavioral health screening tool] as part of an internal pay for performance program. Clinical missed opportunities reports for [behavioral health screening] were developed and tested prior to the end of mid year and rolled out to clinics to drive improvement work.* (Hospital 8, Year 5 Midyear)

Hospitals described efforts to train providers and staff in documentation and billing processes. While considered essential to supporting the sustainability of SBIRT processes, limitations of EHR documentation systems and billing codes were noted:

*While [Hospital] currently participates in administering several of the screening tools referenced within the specification manual (e.g. AUDIT, DAST), the corresponding CPT and HCPC codes have never previously been utilized or captured via billing/claims … infrastructure and operational planning will revolve around the implementation and capture of corresponding coding data at the point of care … so that these endeavors can be sustained beyond the PRIME Waiver.* (Hospital 4, Year 2 Year-end)

#### Restructuring

SBIRT implementation required revisions to professional roles within the hospitals, including hiring providers and staff dedicated to addressing behavioral health and substance use issues and expanding the capacity of primary care providers to conduct brief intervention and utilize medication-assisted treatment. All hospitals in the sample participated in a project within PRIME supporting the integration of behavioral health providers within multidisciplinary care teams within primary care settings, which was integral to the implementation of SBIRT:

*[The system] hired twelve (12) new triad teams consisting of a clinical therapist, a RN care manager and a care coordinator. The teams are co-located in each of the primary care clinics and have obtained a caseload of at least 150 unique patients with behavioral health and complex chronic physical health conditions.* (Hospital 7, Year 2 Year-end)

Some hospitals noted the difficulty of hiring and retaining behavioral health providers and staff, which served as a major barrier to addressing substance use issues*.* Other efforts to expand treatment capacity included the opening of new treatment sites or expansion of spaces for behavioral health treatment. In a few hospitals, the lack of treatment capacity became a deterrent to increasing screening efforts:

*Primary care clinical leaders have been reluctant to start a new screening program that will certainly identify patients with untreated depression and substance use problems without a system in place to offer timely onsite treatment.* (Hospital 16, Year 2 Midyear)

Integrating SBIRT processes into existing clinical workflows was a major focus and challenge amongst competing demands and limited time during visits to identify and address substance use issues:

*… some providers have learned how to bypass this screening when they have a more complicated visit or they do not have a sufficient amount of time to address all of the patients needs …. We are working with the providers to have them document why they skip any given screening to better understand how to improve their workflow. (*Hospital 19, Year 5 Year-end)

Hospitals emphasized the importance of integrating SBIRT data capture and screening tools into the EHR. Tools and functions to facilitate SBIRT processes included templates for SBIRT screening, automatic scoring of substance use measures, alerts for overdue screens, electronic decision support to facilitate follow-up on positive screens, and standardized coding/billing templates. Challenges to integrating EHR tools often hindered hospitals in their attempts to routinize SBIRT processes.

#### Managing quality

Tools developed by hospitals for monitoring SBIRT implementation quality included dashboards and performance reports. Many hospitals described auditing and feedback processes for providers and staff, including workflow observations, generating monthly reports on screening rates, conducting manual chart review, reporting progress at provider/staff meetings, conducting Plan-Do-Study-Act (PDSA) cycles, and having clinic leadership hold providers and staff accountable for implementing SBIRT processes. Tracking of SBIRT performance helped prompt some hospitals to reexamine their implementation strategies:

*A monthly report with the number of new SBIRTs done by clinic is reviewed to track improvement. PRIME metric performance data is emailed to project workgroup leaders each month. Project workgroup leaders review metric numerator fallouts and take appropriate action to improve performance, making sure patients are screened, as appropriate.* (Hospital 6, Year 5 Midyear)

#### Attending to policy context

Some hospitals described implementing complementary policies, such as increasing the number of providers with certification for medication-assisted treatment. Given the focus of the narrative reports on PRIME implementation, efforts to address the external policy context did not emerge as a prominent theme in narratives.

## Discussion

Among a sample of 22 public hospitals in California participating in a five-year value-based program, this study found that hospitals made substantial progress in implementing standardized SBIRT processes within primary care, with wide variation in implementation of screening, brief intervention, and referral to treatment.

Trajectories and paces for implementing SBIRT processes varied, with some hospitals experiencing major delays and others making rapid progress. In many hospitals, more apparent progress occurred in the third year, demonstrating the significant groundwork necessary to implement complex evidence-based practices such as SBIRT within primary care practice. The trajectories of most DMPHs, which often have fewer primary care facilities and smaller patient populations than DPHs, reflected more rapid standardization of SBIRT than DPHs [34–36]. However, challenges prominent in rural healthcare settings such as medical workforce shortages and lack of accessible treatment options hindered timely SBIRT implementation in these settings [56]. County-operated DPHs showed wide variation in SBIRT trajectories, which may correspond to variation in size, geographic locations, and patient populations served by County-operated hospitals.

Findings revealed the most common strategies hospitals employed in implementing SBIRT, with a strong emphasis on strategies for planning, education, and restructuring that were instrumental to their progress. In this study, establishing strong leadership, buy-in, and collaboration among care teams was often influential to initiating and expanding standardized SBIRT processes [19, 20, 22–24, 26, 27]. Consistent with prior research, hospitals described the importance of addressing stigma among providers and staff about substance use through emphasis by leadership, increased training, and deliberate efforts to affirm the importance of SBIRT as a strategy to improve chronic disease outcomes [22, 24, 27]. Planning SBIRT implementation often involved piloting to support the implementation of feasible, adaptable, and effective processes, but may have contributed to delays in rolling out standardized SBIRT processes.

Prior research supports the importance of promoting education of providers to promote SBIRT implementation [19, 20, 23, 26]. In particular, adequate training may address the challenges of discomfort expressed by providers and staff in implementing SBIRT processes, particularly screening and brief intervention, also noted in this study [22, 24]. Novel strategies to engage providers and staff in SBIRT training efforts described by hospitals in this study included the use of post-training tests to gauge learning, and financial incentives for those completing SBIRT training programs. Prior studies have highlighted the difficulties of implementing workflow changes in clinical settings, including challenges to unlearning existing practices and promoting adherence to clinical guidelines [57, 58]. Given these difficulties, hospitals in this sample may have leveraged program incentives to drive practice change.

The findings of this study also highlighted intensive efforts to restructure existing infrastructure and processes in implementing SBIRT. Consistent with prior studies, hospitals discussed extensive efforts to incorporate SBIRT into clinic workflows, often in the face of challenges such as limited provider/staff buy-in or comfort with SBIRT processes and lack of capacity for substance use treatment [19, 20, 22, 24, 26]. For many hospitals, EHR integration of SBIRT processes was a key driver of systematic and standardized SBIRT processes but was often difficult to implement in a timely manner, leading to delays in standardizing their SBIRT processes. However, hospitals able to overcome this hurdle developed and leveraged tools within the EHR to facilitate the implementation of SBIRT within their primary care settings.

### Limitations

The study has several limitations to note. First, the use of hospital-level data limited the ability to directly assess specific perspectives of system providers, clinical support staff, and other staff within the health systems (e.g., information technology staff) involved in the implementation of SBIRT. However, key informants who completed the narrative reports included hospital leaders intimately familiar with provider and staff perspectives, workflows, and organizational priorities. While the use of narrative reports required for participation in the PRIME program allowed for the longitudinal assessment of SBIRT implementation outcomes without additional reporting burden, there was variation in the comprehensiveness and content of narratives between hospitals. Additionally, we used narrative data to assess SBIRT reach and did not assess this implementation outcome quantitatively. As provider adoption of SBIRT was not a focus of the narrative report templates, we were unable to systematically assess this outcome. Additionally, narratives likely excluded information that hospital leadership may not have wanted to report to administrators and evaluators of the PRIME program. Our sample included public hospitals within California participating in a value-based program. While our findings may be relevant for other safety net healthcare settings (e.g., public hospitals outside of California, federally qualified health centers), the findings may not be generalizable to other settings. Finally, analysis of trajectories of SBIRT implementation assumed the sequential implementation of standardized processes for each step of SBIRT.

### Implications for policy and practice

This study contributes to the evidence base surrounding strategies for implementing SBIRT in public hospitals, including County-operated, academic, and rural hospitals which often serve as safety net healthcare settings for a wide population of patients [5–14, 59, 60]. Public hospitals serve as an important provider of care for patient populations with higher complexity health needs, underscoring the importance of effective use of evidence-based practices to address unhealthy use of alcohol and other substances.

Difficulties with electronic health record integration and behavioral health staffing highlighted key areas in which additional support or investment may be needed to promote and sustain long-term progress in SBIRT implementation. Specific modes of implementation support, such as practice facilitation, have been shown to promote SBIRT within low-resource healthcare settings [29]. Significant improvements in the capacity of EHRs to support SBIRT processes (e.g., development of standardized EHR screening templates, clinical decision support, documentation tools) and guidance on best practices for integrating SBIRT processes within EHRs may help facilitate SBIRT implementation in primary care settings [61–63].

Many hospitals in this study noted leveraging external sources of funding, highlighting the high level of resources and effort required to address substance use and underscoring the importance of allocating financial support to drive SBIRT implementation. Additionally, low reimbursement rates and challenges with standardizing SBIRT documentation remain barriers to obtaining reimbursement for SBIRT [64, 65]. Expanding the range of reimbursable services, providing health systems with guidance and technical assistance in obtaining reimbursement, and improving reimbursement rates for SBIRT processes may improve implementation and sustainability [64, 66].

This study highlighted considerable variation in the trajectories and strategies utilized by public hospitals participating in the same value-based program in implementing SBIRT. Some hospitals, particularly those with large primary care networks, may prefer to tailor or pilot SBIRT workflows to accommodate variation in clinic and patient characteristics across sites. However, increased standardization of guidance for implementing SBIRT (e.g., identifying specific evidence-based screening tools to implement) within value-based programs and from guiding bodies may help expedite implementation [29]. Prior research has identified SBIRT-related disparities, including a greater rate of discordance between need and receipt of SBIRT services among older and Hispanic adults [67]. Promoting standardization in the use of best practices for SBIRT may help promote equity in the receipt of appropriate SBIRT services.

In evaluating SBIRT initiatives, improvements to data collection (e.g., structured fields, validated assessment tools) may facilitate more comprehensive assessment of progress and challenges in implementing SBIRT and related interventions. Building a clearer understanding of the implementation trajectories, strategies, and challenges can help government agencies and other guiding bodies support health systems in their implementation of SBIRT and other interventions to address substance use.

## Conclusions

This study highlighted significant progress and variation in strategies used by public hospitals in implementing SBIRT in primary care within a value-based program. However, hospitals experienced delays and challenges, highlighting key areas in which additional support or investment may be needed to sustain and promote long-term progress in SBIRT implementation.

## Data Availability

The data supporting the conclusions of this article were obtained from a third party (California Department of Health Care Services), and may be available upon request.
